# Comparative effects of dietary sodium butyrate and tributyrin on broiler chickens’ performance, gene expression, intestinal histomorphometry, blood indices, and litter

**DOI:** 10.1038/s41598-025-09278-3

**Published:** 2025-07-18

**Authors:** Elshaimaa Ismael, Shaimaa Kamel, Ebtihal M. M. Elleithy, Manal R. Bekeer, Khaled Nasr El-din Fahmy

**Affiliations:** 1 Department of Veterinary Hygiene and Management, Faculty of Veterinary Medicine, Cairo University, PO Box 12211, Giza, 12211 Egypt; 2 Department of Biochemistry and Molecular Biology, Faculty of Veterinary Medicine, Cairo University, Giza, 12211 Egypt; 3 Department of Cytology and Histology, Faculty of Veterinary Medicine, Cairo University, Giza, 12211 Egypt; 4 Department of Physiology, Faculty of Veterinary Medicine, Cairo University, Giza, 12211 Egypt; 5 Department of Nutrition and Clinical Nutrition, Faculty of Veterinary Medicine, Cairo University, Giza, 12211 Egypt

**Keywords:** Glycerol esters of butyric acid, Lipase activity, *Clostridia*, *mTOR*, *TLR4*, Jejunal villi, Animal physiology, Antimicrobials, Analytical biochemistry, Gene expression analysis, Microscopy

## Abstract

Sodium butyrate and tributyrin are known to enhance broiler chicken performance. In this study, 1,000 Arbor Acres broiler chicks were assigned to four dietary treatments (250 birds each; six replicates of 40–42 birds): a control basal diet (CON), or the same diet supplemented with either 500 g/ton tributyrin (40%) + copper + essential oils (TB-500), 300 g/ton di- and tri-butyrin (60%) (TB-300), or 500 g/ton coated sodium butyrate (40%) (SB-500). Weekly growth parameters were recorded, and on Day 35, carcass traits, serum biochemistry, immunity, gene expression (*mTOR, TLR4, NBN*), intestinal morphology, caecal microbiota, and litter hygiene were assessed. TB-300 improved body weight (+ 4.6%, *P* = 0.014), FCR (− 5.2%, *P* = 0.032), and European Production Efficiency Factor (EPEF) (+ 14.9%, *P* = 0.006). SB-500 significantly reduced litter *Clostridia* (*P* < 0.0001) and aerobic bacteria (*P* = 0.026) counts, while all butyrate treatments lowered caecal aerobic bacterial levels (*P* = 0.041). TB-300 and SB-500 enhanced duodenal villi height (*P* < 0.0001) and crypt-villus ratio (*P* < 0.001); TB-500 had the deepest duodenal crypts (*P* = 0.003). Jejunal and ileal morphology improved with most of the supplements, particularly TB-500 (*P* < 0.0001; *P* = 0.050). All butyrate treatments increased serum total proteins (*P* = 0.015) and digestive enzymes (lipase, *P* < 0.0001; protease, *P* = 0.001). TB-300 and SB-500 significantly lowered serum lipids (*P* = 0.024), urea (*P* = 0.018), and aspartate aminotransferase (AST) (*P* = 0.027), while enhancing *mTOR* and *NBN* gene expression (*P* < 0.0001). *TLR4* expression was upregulated in all butyrate-treated groups (*P* < 0.0001). Each form of butyrate supplementation exerts distinct beneficial effects on growth, gut health, and physiological performance in broiler chickens.

## Introduction

Organic short-chain fatty acids (SCFAs)—such as propionic, acetic, and butyric acids—are primarily produced in the lower intestinal tract through anaerobic microbial fermentation and serve as vital energy sources for colonocytes^1^. SCFAs are increasingly used as feed additives to replace antibiotics previously employed as growth promoters^2,3^. Among them, butyric acid is a key volatile SCFA with numerous applications in the animal feed industry. Butyrate and its derivatives have demonstrated preventive and therapeutic effects against intestinal and colonic diseases. Notably, butyric acid can activate regulatory T cells in the colon, which help suppress inflammatory and allergic responses^4^. However, the use of butyric acid as a feed additive poses challenges due to its unpleasant odor and rapid absorption in the upper digestive tract, limiting its effectiveness in the field^1,5^. Various butyrate formulations have been developed to mask its odor and delay its absorption until it reaches the intestine, thereby improving gut health, mitigating digestive disorders, and enhancing bird performance^1,2^.

Butyric acid salts are commonly used as growth promoters; however, their effectiveness can be inconsistent due to factors such as dosage, duration of application, physical form, and the age of the animals^3,6,7^. Sodium butyrate (SB), a widely used dietary butyrate supplement, reaches the intestine and supports gut barrier function, increases SCFA levels, and positively influences the microbiota. To enhance its stability and ensure effective intestinal delivery, coating technologies using buffer salts have been developed. Several studies have evaluated the effects of dietary supplementation with 600–1000 mg/kg of coated SB in broiler feed and observed improvements in body weight gain and feed conversion ratios^8,9^. However, some studies have reported no significant impact on performance^10^. Despite these mixed results, there is general agreement on the beneficial role of long-term administration of coated SB in modulating gut microbiota toward more favorable bacterial populations^9,10^.

Another common derivative of butyrate is tributyrin (TB), which offers beneficial but distinct effects on broiler growth and performance^11^. TB is a tri-ester form of butyric acid that undergoes rapid hydrolysis by intestinal lipase, producing mono-butyrin and butyric acid, which are then quickly absorbed in the small intestine^12^. Previous studies have highlighted several advantages of TB over SB, including a longer metabolic half-life, the ability to induce host defense peptides, and a more acceptable odor^13–15^. While butyric acid is rapidly cleared from the body within 2–3 h due to its rapid cellular uptake as a preferred energy source, TB has a longer half-life in the blood, approximately 4 h, which is about 40 min longer than that of butyric acid^5^. Therefore, using butyrate derivatives, particularly glycerol esters like tributyrin, can help overcome the undesirable physical and biochemical properties of free butyric acid. Butyrate glycerides, including mono-, di-, and tri-butyrin, are formed by attaching varying numbers of butyric acid molecules to a glycerol backbone. Once in the small intestine, lipase enzymes hydrolyze the glycerol ester, releasing butyrate, which remains shielded from absorption in the upper gastrointestinal tract^1^. Glycerol esters of butyrate are currently used as feed additives because of their superior characteristics compared to their parent SCFAs, including being non-corrosive, non-volatile, heat-stable, and possessing antibacterial properties. Unlike the pungent odor of butyric acid, these esters have a more neutral and pleasant fruity aroma, often compared to the scent of apples or pineapples^5^.

Recently, mono- and tri-butyrin have been increasingly used as dietary supplements in poultry due to their positive effects on growth performance, intestinal mucosal integrity, and antimicrobial activity against pathogens such as *Clostridium perfringens* and *Salmonella Typhimurium*. In addition, these compounds have been shown to reduce abdominal fat and serum lipid levels by enhancing fatty acid oxidation in the liver and decreasing lipid synthesis, storage, and transport in the jejunum^5,11^. Additionally, butyrate may influence gene expression in skeletal muscle and immune tissues, thereby affecting muscle growth, metabolism, and immunity in broilers. SB has been shown to modulate the expression of the mammalian target of rapamycin (*mTOR*), a key regulator of cell growth, in mouse adipose tissue^16^. Butyrate supplementation has also been associated with upregulation of Toll-like receptor 4 (*TLR4*), a gene involved in innate immunity, in certain cell types^17^. The *NBN* gene encodes nibrin, a protein essential for DNA repair and genomic stability, particularly in rapidly dividing muscle and immune cells, and may also be influenced by butyrate. Although direct evidence in broilers is limited, butyrate’s function as a histone deacetylase inhibitor^18^ suggests it could modulate *NBN* expression and associated DNA repair pathways.

This study evaluated the effects of three butyrate-based feed additives—tributyrin with copper and essential oils (TB-500), di and tributyrins (TB-300), and coated sodium butyrate (SB-500)—supplemented in a corn-based basal diet, on broiler chickens. Parameters assessed included growth performance, carcass traits, serum biochemistry, digestive enzyme activity, intestinal histomorphometry, cecal microbiota, litter quality, and the expression of growth- and immunity-related genes (*mTOR* in muscle; *TLR4* and *NBN* in liver). The comparative analysis of these formulations offers insight into their potential to enhance broiler health and performance.

## Methods

### Ethical approval

All experimental procedures in this study were approved by the Institutional Animal Care and Use Committee of the Faculty of Veterinary Medicine, Cairo University, Egypt (Approval Reference No.: Vet CU28/04/2021/279). All methods were carried out in accordance with relevant guidelines and regulations, and the study is reported in compliance with the ARRIVE guidelines.

### Housing of birds

The current experiment was conducted at the Center of Animal and Poultry Research, Faculty of Veterinary Medicine, Cairo University, Giza, Egypt, and lasted for 35 days. A total of 1,000 day-old broiler chicks (Arber-Acres) were purchased from a commercial hatchery, with an average initial weight of 47 g. The chicks were randomly assigned to four treatment groups (250 birds per treatment), each subdivided into six replicates (pens), with 40–42 birds per replicate. The birds were housed in separate pens (2.7 m in length × 1.4 m in width), which were constructed with concrete floors and bedded with clean wood shavings (10 cm depth). The house was semi-closed, equipped with exhaust fans and cooling pads for ventilation. Feed and water were continuously available in manual feeders and drinkers. The house temperature was maintained at 33 °C for the first three days of age, with a weekly decrease of 2.8 °C until it reached 24 °C, which was maintained for the duration of the experiment. The relative humidity inside the house was maintained at approximately 55–60% throughout the experiment. For the first three days, the birds were exposed to continuous light, followed by a 23-h light/1-h dark cycle for the remainder of the experiment. Vaccinations were administered via eye drops against Newcastle disease virus (NDV-Hitchner B1) on Day 6 and NDV-Lasota on Day 18. Additionally, birds were vaccinated against Infectious Bursal Disease and Avian Influenza (H5N1) on Day 14 through subcutaneous injection (0.2 ml per bird).

### Experimental design

The birds were fed a formulated corn-soybean meal-based basal diet, which met the nutritional requirements for broilers^19^. Birds had unlimited access to feed and water throughout the study. The broiler chicks were provided with a starter crumble diet (days 1–14), followed by a grower pelleted diet (days 15–27), and a finisher pelleted diet (days 28–35) (Table 1). On Day one, the broiler chickens were randomly assigned to one of four dietary treatments (250 birds per treatment), with each treatment replicated six times, resulting in 40–42 birds per replicate (pen). The four dietary treatments were as follows: a control basal diet without anti-clostridial feed additives (CON), and three experimental diets supplemented with different additives: (1) **TB-500:** 500 g/ton of Villa-Guard P™ (Solveda, Egypt) [40% glycerol esters of butyric acid (tributyrin) + 2.5% copper oxin + essential oils (thymol 1% and allicin 1.5%) + 0.5% saponin], providing 200 g tributyrin/ton of feed; (2) **TB-300**: 300 g/ton of ProPhorce™ SR 130 (Perstorp Waspik BV, Netherlands) [60% glycerol esters of butyric acid (di- and tributyrin)], providing 180 g di- and tributyrin/ton of feed; (3) **SB-500**: 500 g/ton of Adimix® Precision (Adisseo, France) [40% sodium butyrate (SB) coated with 60% palm oil], providing 200 g SB/ton of feed. The feed additives and supplements were initially blended in a micromixer to ensure uniform distribution. Subsequently, they were incorporated into the feed ingredients during the final mixing process using the horizontal mixer at the feed mill.

**Table 1:** Physical ingredients and chemical analysis of basal broiler diets during different rearing phases (1 to 35 days of age).

Composition	Starter (0–14 d)	Grower (15–28 d)	Finisher (29–35 d)
Physical ingredients (%):			
Yellow corn	55.24	59.39	63.64
SBM 46% CP	27.00	18.60	10.30
Full-fat SBM	8.00	12.50	16.00
Corn-gluten meal 60% CP	6.00	6.00	6.50
Monocalcium phosphate	0.90	0.80	0.80
Limestone	1.60	1.50	1.50
Sodium chloride	0.35	0.35	0.35
Sodium bicarbonate	0.10	0.10	0.10
L-Lysine	0.25	0.25	0.30
DL-Methionine	0.15	0.10	0.10
Toxin binder	0.10	0.10	0.10
Quantum blue 5 G (Phytase)	0.01	0.01	0.01
Broiler premixes^1^	0.30	0.30	0.30
Chemical analysis:			
ME (Kcal/kg)	3001.05	3100.63	3200.24
CP (%)	23.17	21.11	19.14
Crude Fat (%)	3.96	4.87	5.60
Calcium (%)	1.00	0.94	0.93
P Available (%)	0.50	0.45	0.42

SBM: Soybean meal, CP: crude protein, ME: metabolizable energy, P: phosphorus.

^1^Mineral mixture: manganese oxide (120,000 mg); zinc oxide (100,000 mg); copper sulphate (15,000 mg); calcium iodide (1000 mg); ferrous sulphate (50,000 mg); selenium selenite (350 mg).

^1^Vitamin mixture: vit. A (13,000,000 IU); vit. D_3_ (6,000,000 IU); vit. E (80,000 mg); vit. K (4000 mg); vit. B1 (5000 mg); vit. B2 (9000 mg); vit. B6 (5000 mg); vit. B12 (35 mg); pantothenic acid (20,000 mg); nicotinic acid (70,000 mg); folic acid (2000 mg); biotin (250 mg); choline chloride (400,000 mg).

### Growth performance

Body weight (BW), feed intake (FI), and mortality rates were recorded weekly. Total feed consumption per week was divided by the number of birds in each treatment group, adjusted for mortality, to calculate the average weekly feed intake per bird. Feed conversion ratio (FCR) and the European Production Efficiency Factor (EPEF) were also calculated. The feed conversion ratio (FCR) was determined by dividing the weekly feed intake by the weekly weight gain, accounting for the number of dead birds. The European Production Efficiency Factor (EPEF) was calculated using the following formula ^20^:

$$EPEF =\frac{Body weight (kg) \times \% Liveability }{Feed conversion ratio \times Age of birds in days} \times 100$$

### Carcass traits

On day 35 of the experiment, after a 4-h fasting period to ensure complete gut evacuation, six birds from each treatment were selected, weighed, and humanely slaughtered by competent personnel through severing both jugular veins, both carotid arteries, and the trachea at the neck using a sharp knife, to ensure rapid loss of consciousness and effective exsanguination, following the American Veterinary Medical Association (AVMA) Guidelines for the Euthanasia of Animals^21^. The birds were scalded, de-feathered, and eviscerated after the removal of the head, neck, and legs. The carcass weight, excluding giblets, was recorded and expressed as a percentage of the live weight (carcass yield). The carcasses were then dissected, and the weights of the breast, drumstick, and thigh were measured. The relative weights of these body parts were calculated as a percentage of the total carcass weight.

### Sampling of litter, cloacal, and caecal content

From each treatment, six samples were collected, where litter samples were obtained on Days 23 and 35. The top 7 cm of deep litter was scooped from three different locations within each pen and placed in sterile plastic bags^22,23^. Cloacal swabs were collected on Day 23 and placed into tubes containing 5 ml of sterile saline^24^. Additionally, caecal samples were collected after the birds were sacrificed on Day 35. All samples were stored at 4°C until further bacteriological analysis.

### Microbiological examinations of litter, cloacal, and caecal content

For microbial analysis of litter samples, the litter was diluted in sterile saline solution at a ratio of 1:10 to prepare a 10^−1^ dilution. The samples were kept at room temperature for 30 to 60 min and shaken frequently to ensure thorough mixing of the litter with the diluent^22,23^. One milliliter of cloacal swab sample and one gram of caecal content were diluted in 9 ml of sterile saline solution and homogenized (10^−1^ dilution)^25^. The samples were then subjected to a tenfold serial dilution in tubes containing 9 ml of sterile saline solution. From the serially diluted tubes, 100 µl of each sample was spread onto Nutrient Agar and Reinforced Clostridial Agar (Oxoid Ltd, Basingstoke, Hants, UK) to enumerate total aerobes, total anaerobes, and *Clostridia*, respectively. All plates were incubated at 37 °C for 24 to 48 h, with tightly sealed anaerobic jars used to maintain anaerobic conditions. Finally, the bacterial colony counts were reported as the mean 10-logarithmic colony-forming units (log_10_ CFU) per unit of litter, cloaca, and caecal contents.

### Physical and chemical analysis of litter

Litter moisture content was determined following the method previously described^22^. A 10 g subsample of litter from each replicate was weighed and dried in a hot air oven at 100 ± 5 °C for 24 to 48 h. Moisture content was calculated by subtracting the dry weight from the initial sample weight. Total nitrogen content in the litter was analyzed using the Kjeldahl method, expressed as total Kjeldahl nitrogen^26^.

### Histological examination

#### Light microscopy

On day 35 of the experiment, the small intestines were collected from five birds per treatment. Intestines were excised, opened longitudinally, and gently flushed with 0.1 M phosphate-buffered saline (pH 7.4). Sections from the middle of the duodenum, jejunum (at the midpoint between the bile duct entry and Meckel’s diverticulum), and ileum (about 0.5 cm in length) were fixed immediately in a 10% neutral buffered formalin solution. Following fixation in formalin, the tissue samples were processed through dehydration in ascending concentrations of ethanol (50%, 70%, 80%, 90%, and absolute alcohol), followed by clearing in an intermediate solvent—xylene—that is miscible with both alcohol and paraffin. The samples were cleared with two changes of xylene, then infiltrated and embedded in three changes of paraffin wax to form paraffin blocks. These blocks were subsequently sectioned using a microtome. To verify general histological structural features, 6–7 mm-thick sections were prepared, mounted on clean glass slides, and stained with Delafield’s iron Haematoxylin and Eosin^27^.

#### Histomorphometry of the small intestines

Histomorphometric analysis was conducted to measure villus length and crypt (intestinal gland) depth in the mucosa of the small intestine. Stained sections were analyzed using a Leica Quin 500 image analyzer system (Leica Microsystems, Switzerland). The system was automatically calibrated to convert measurement units from pixels to micrometers. Images of each section were captured at a final magnification of 40X.

### Biochemical analysis

On day 35 of the experiment, blood samples were collected from five birds/treatment after being slaughtered. The blood was collected into plain tubes, centrifuged at 1000×g for 20 min, and serum samples were stored at – 20 °C until analysis. Serum samples were assayed for amylase, protease, and lipase activities^28–30^. The serum total protein was determined according to^31^, while albumin was measured as described by^32^. Total lipids, cholesterol, and triglycerides were measured spectrophotometrically according to the methods described by^72^ . Serum urea and creatinine concentrations were measured spectrophotometrically^33,34^. Serum alanine aminotransferase (ALT) and aspartate aminotransferase (AST) activities were estimated as described by ^73^. The serum biochemical parameters were measured using enzymatic spectrophotometry as recommended by the manufacturer’s procedure (Spinreact, Spain).

### Vaccinal antibody levels against Newcastle disease virus (NDV)

The haemagglutination inhibition (HI) test was performed to evaluate the vaccinal antibody titers against NDV^35^. Briefly, 25 µl of each serum sample was two-fold serially diluted in 96-well microplates and tested against 25 µl of four haemagglutination units of NDV-LaSota commercial antigen. After gentle mixing, the plates were incubated at room temperature for 20 min to allow antigen–antibody interaction. Subsequently, 25 µl of a 1% suspension of packed chicken red blood cells was added to each well. HI titers were recorded after a 30 to 45-min incubation, and results were expressed as log_2_ values.

### Genetic qRT-PCR analysis of *mTOR*, *TLR4*, and *NBN* genes

On day 35 of the experiment, liver tissue, breast muscle, and thigh muscle samples were collected from five birds per treatment for gene expression analysis. Total RNA was extracted from 100 mg of each sample using the easy-spin Total RNA Extraction Kit, following the manufacturer’s instructions (Cat. No. 17221; iNtRON Biotechnology, South Korea). The concentration and purity of the RNA were assessed using a NanoDrop ND-1000 spectrophotometer^36^. Complementary DNA (cDNA) was synthesized using M-MLV Reverse Transcriptase (Enzynomics, Cat. #RT001S) according to the manufacturer’s protocol. The 20 μl reverse transcription reaction contained 2 μl of 10 × RT buffer, 1 μl of reverse transcriptase (200 U/μl), 2 μl of dNTP mixture, 50 μg of RNA template, 1 μl of oligo(dT) primer, 0.5 μl of RNase inhibitor (40 U/μl), and RNase-free water to complete the final volume.

Quantitative real-time PCR (qRT-PCR) was performed using the HERA^PLUS^ SYBR Green qPCR kit (Cat. #WF10308002) to evaluate the mRNA expression levels of the *mTOR* gene in breast and thigh muscles, and *TLR4* and *NBN* genes in liver tissues. Each 20 μl reaction mixture consisted of 10 μl of HERA^PLUS^ SYBR Green Master Mix (2X), 1 μl each of forward and reverse primers (20 × , 200 nM), 2 μl of cDNA template, and nuclease-free water. The qPCR cycling conditions were as follows: initial denaturation at 95 °C for 2 min, followed by 40 cycles of denaturation at 95 °C for 10 s and annealing/extension at 60 °C for 30 s. All reactions were run in triplicate^37^. The housekeeping gene *β-actin* was used as an internal control^38^. Primers were designed using the Primer3 software (https://primer3.ut.ee/), and the sequences are presented in Table 2. Gene expression data were analyzed using the ΔCT, ΔΔCT, and 2^−ΔΔCT^ methods^39^.

**Table 2:** Primer sequences used for the qRT-PCR.

Gene symbol	Gene description	Accession number	Amplicon size	Primer Sequence
*mTOR*	Mechanistic Target of Rapamycin	XM_417614.6	213	F: 5′-CGCAGTGAAGAAACAAGGGC-3′ R: 5′-GGTGGCGTTACCTCCTTCAA-3′
*NBN*	Nibrin	NM_204337.1	159	F: 5′-GCTTGGAAGGGAAAGTGGTG-3′ R: 5′-TCCCAGTCTAGGTCTCTGCT-3′
*TLR-4*	Toll-like receptor 4	NM_001030693.1	158	F: 5′-ATGTCCTCTTGCCATCCCAA-3′ R: 5′-TCTCCCCTTTCTGCAGAGTG-3′
*β-actin*	Beta-actin	L08165.1	177	F: 5′-CCCACACCCCTGTGATGAAA-3′ R: 5′-TAGAACTTTGGGGGCGTTCG-3′

### Statistical analysis

Results were reported as means ± standard error of the means (SEM). A linear mixed-effects model was used to assess the effects of treatment (CON, TB-500, TB-300, SB-500), time (Day 7 to Day 35), and their interaction on body weight (BW), feed intake (FI), feed conversion ratio (FCR), and bacterial counts, with pen included as a random intercept to account for repeated measures. The analysis was conducted using the *lme4*, *lmerTest*, and *emmeans* packages in R (Version 4.4.3)^40–42^. Pairwise comparisons were performed using estimated marginal means with Tukey’s adjustment for multiple testing and Kenward-Roger approximation for degrees of freedom. For all other parameters, one-way analysis of variance (ANOVA) was conducted, followed by Tukey’s post hoc test for multiple comparisons, using PASW Statistics software, Version 18.0 (SPSS Inc., Chicago, IL, USA). Statistical significance was set at *P* ≤ 0.05. Box and bar plots were generated using the *ggplot2* package in R^43^.

## Results

### Weekly growth performance

There was a significant time × treatment interaction for broiler BW (*P* < 0.0001), indicating that the effect of dietary treatments varied across the different time points (Table 3). At Day 28, birds in the SB-500 group showed significantly higher body weight compared to the CON (+ 78.0 g, *P* < 0.0001), TB-300 (+ 55.8 g, *P* = 0.005), and TB-500 (+ 56.0 g, *P* = 0.005) groups. Similarly, at Day 35, SB-500 (+ 49.6 g, *P* = 0.017), TB-300 (+ 86.8 g, *P* < 0.0001), and TB-500 groups (+ 66.8 g, *P* < 0.001) birds maintained significantly higher BW than the CON. No significant differences in body weight were observed among groups at earlier time points (Day 7 to 21) (*P* > 0.05). These results suggest that the effect of butyrate supplementation became more evident in later growth stages.

**Table 3:** Impact of different dietary butyrate formulations on weekly performance of broiler chickens through Days 7 to 35.

Groups	Body Weight (g)					Feed Intake (g)					FCR (g/g)				
	D 7	D 14	D 21	D 28	D 35	D 7	D 14	D 21	D 28	D 35	D 7	D 14	D 21	D 28	D 35
CON	177	467	945	1502^b^	1893^b^	141	346	608	751	1011	1.09	1.20	1.28	1.36	2.68^a^
TB-500	177	464	932	1524^b^	1960^a^	140	350	590	788	998	1.08	1.22	1.27	1.30	2.28^b^
TB-300	180	463	952	1524^b^	1980^a^	142	349	633	749	965	1.07	1.24	1.29	1.32	2.10^b^
SB-500	178	468	919	1580^a^	1943^a^	140	349	613	776	998	1.08	1.20	1.37	1.18	2.72^a^
SEM^1^	1.30	3.34	6.03	8.62	10.56	0.91	1.72	6.23	6.77	6.79	0.01	0.01	0.02	0.03	0.09
*P* (Group)	0.011					0.692					0.040				
*P* (Time)	< 0.0001					< 0.0001					< 0.0001				
*P* (Int.)	< 0.0001					0.002					< 0.001				

^a,b^Means showing variable letters within the same column denote statistical significance (Tukey-adjusted; *P* ≤ 0.05).

**CON**, Control basal diet; **TB-500**, basal diet + tributyrin 40%/copper/essential oils “Villa-Guard P™” (500g/ton feed); **TB-300**, basal diet + butyrins glycerol esters 60% “ProPhorce™ SR 130” (300g/ton feed); **SB-500**, basal diet + coated sodium butyrate 40% “Adimix® Precision” (500g/ton feed).

FCR, Feed Conversion Ratio (g of feed / g of weight gain).

^1^SEM: Pooled standard error of the mean.

N = 10 birds per replicate (pen), (60 birds per treatment).

A significant interaction between treatment group and time was detected for FI (*P* = 0.002) (Table 3). No significant differences in FI were observed among groups during Day 7 and Day 15 (*P* > 0.05). At Day 21, TB-300 showed a significantly higher FI compared to TB-500 (+ 43.0, *P* = 0.011). At Day 28, the TB-500 group had significantly higher FI compared to CON (+ 36.8, *P* = 0.038) and TB-300 (+ 39.5, *P* = 0.022). At Day 35, TB-300 had significantly lower FI than the CON group (− 45.8, *p* = 0.006), while differences between the other groups were not statistically significant (*P* > 0.05).

There was a significant interaction between group and time for FCR (*P* < 0.001) (Table 3). No significant differences in FCR were observed between groups during Days 7 to 28 (*P* > 0.05). At Day 35, the TB-300 and TB-500 groups had significantly lower FCR compared to CON (TB-300: − 0.58, *P* < 0.0001; TB-500: − 0.40, *P* = 0.002). Additionally, the TB-300 and TB-500 groups had significantly lower FCR compared to SB-500 (TB-300: − 0.62, *P* < 0.0001; TB-500 (− 0.44, *P* < 0.001).

### Cumulative growth performance

Cumulative performance data are summarized in Table 4. Final BW was significantly higher in the TB-300 group compared to the CON (*P* = 0.014). FCR was also significantly improved in the TB-300 group (*P* = 0.032), indicating better feed efficiency. The European Production Efficiency Factor (EPEF) was significantly higher in the TB-300 group than in the CON (*P* = 0.006). No significant differences were observed among groups for FI or mortality (*P* > 0.05).

**Table 4:** Impact of different dietary butyrate formulations on cumulative growth performance of broiler chickens (Days 1–35).

Groups	BW (g)	FI (g)	FCR (g/g)	EPEF	Mortality (%)
CON	1893^b^	2857	1.55^a^	328^b^	6.35
TB-500	1960^ab^	2865	1.50^ab^	349^ab^	6.74
TB-300	1980^a^	2837	1.47^b^	377^a^	2.38
SB-500	1943^ab^	2875	1.52^ab^	343^ab^	6.35
SEM^1^	10.56	11.13	0.01	5.48	0.68
*P*-value	**0.014**	0.684	**0.032**	**0.006**	0.063

Significant values are in bold.

^a,b^Mean showing variable letters within the same column denote statistical significance (Tukey’s test; *P* ≤ 0.05).

**CON**, Control basal diet; **TB-500**, basal diet + tributyrin 40%/copper/essential oils “Villa-Guard P™” (500g/ton feed); **TB-300**, basal diet + butyrins glycerol esters 60% “ProPhorce™ SR 130” (300g/ton feed); **SB-500**, basal diet + coated sodium butyrate 40% “Adimix® Precision” (500g/ton feed).

BW, Body Weight; FI, Feed Intake; FCR, Feed Conversion Ratio (g of feed / g of weight gain).

EPEF: European Production Efficiency Factor = [(livability × BW (kg)) / (age (days) × FCR)] × 100.

^1^SEM: Pooled standard error of the mean.

N = 10 birds per replicate (pen), (60 birds per treatment).

### Carcass traits

Carcass traits are presented in Table 5. No significant differences were observed in dressing percentage, or in the yields of breast, thigh, or drum muscles (*P* > 0.05).

**Table 5:** Impact of different dietary butyrate formulations on carcass traits of broiler chickens (Day 35).

Groups	Dressing (%)	Breast (%)	Thigh (%)	Drum (%)
CON	76.50	30.22	26.90	13.46
TB-500	72.95	31.50	26.89	13.98
TB-300	75.34	30.82	27.59	13.87
SB-500	74.59	30.92	28.12	13.39
SEM^1^	1.23	0.63	0.43	0.20
*P*- value	0.799	0.925	0.725	0.675

Statistical significance was set at *P* ≤ 0.05.

**CON**, Control basal diet; **TB-500**, basal diet + tributyrin 40%/copper/essential oils “Villa-Guard P™” (500g/ton feed); **TB-300**, basal diet + butyrins glycerol esters 60% “ProPhorce™ SR 130” (300g/ton feed); **SB-500**, basal diet + coated sodium butyrate 40% “Adimix® Precision” (500g/ton feed).

^1^SEM: Pooled standard error of the mean.

N = 6 birds per treatment.

### Microbiological counts of litter

**Fig. 1:**
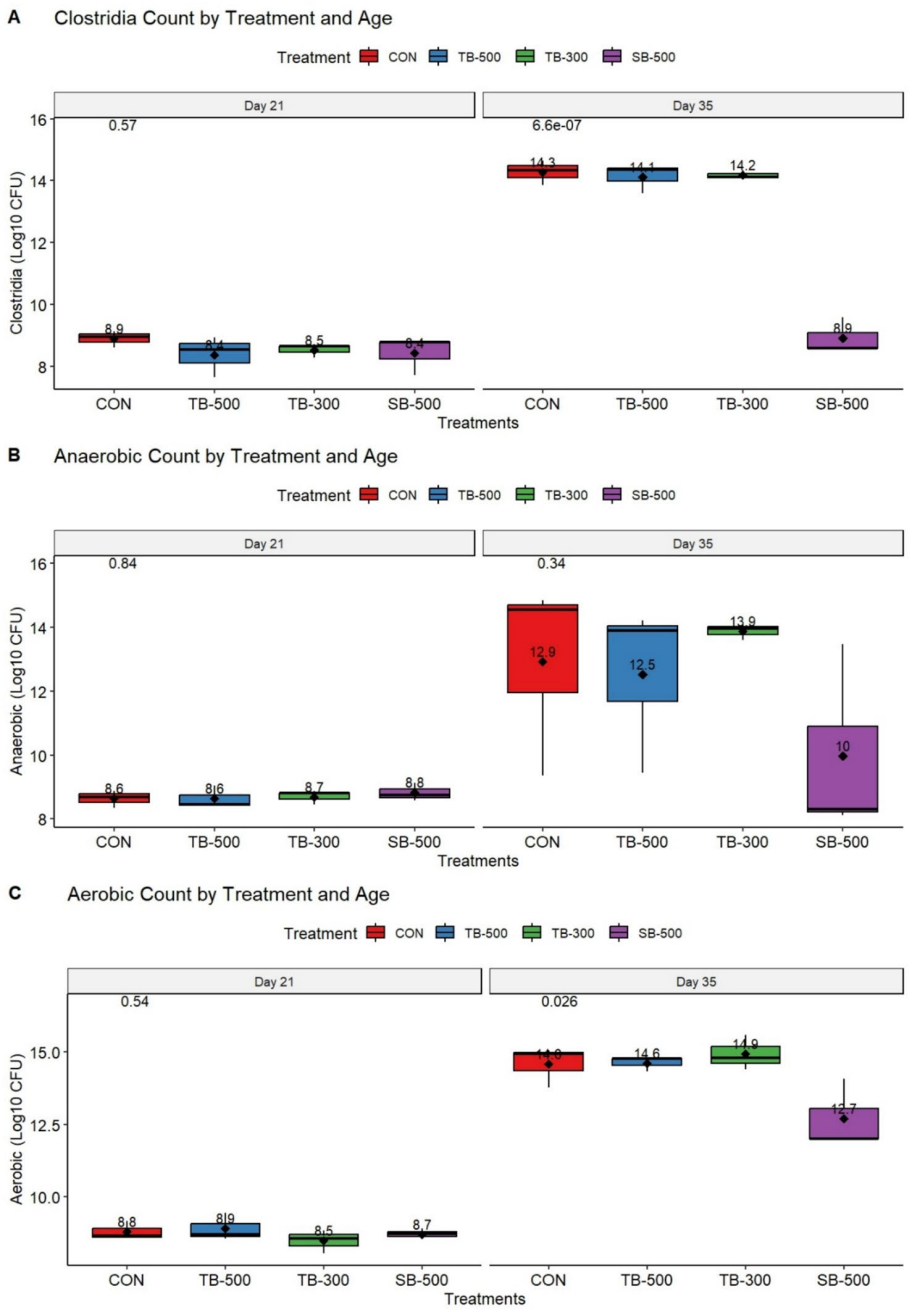
Impact of different dietary butyrate formulations on deep-litter bacterial counts of broiler chickens at Days 21 and 35 of age. **CON**, Control basal diet; **TB-500**, basal diet + tributyrin 40%/copper/essential oils “Villa-Guard P™” (500g/ton feed); **TB-300**, basal diet + butyrins glycerol esters 60% “ProPhorce™ SR 130” (300g/ton feed); **SB-500**, basal diet + coated sodium butyrate 40% “Adimix® Precision” (500g/ton feed). Values of means were displayed above boxplots. Statistical significance was set at *P* ≤ 0.05. N = 6 litter samples per treatment.

Figure 1 illustrates the bacteriological load of deep litter on Days 21 and 35 of the experiment. There was a significant interaction between age and treatment on litter *Clostridia* counts (*P* < 0.0001) (Fig. 1A). On Day 21, no differences were detected among groups (*P* > 0.05).

By Day 35, the SB-500 group had significantly lower *Clostridia* counts compared to the CON group (*P* < 0.0001), while TB-300 and TB-500 did not differ significantly from CON (*P* > 0.05). Within-group comparisons over time showed that *Clostridia* counts increased significantly from Day 21 to Day 35 in the CON, TB-300, and TB-500 groups, exceeding 5 log_10_ CFU (*P* < 0.0001), whereas the SB-500 group maintained stable levels over time (*P* = 0.887).

A significant main effect of age (time) was observed on litter anaerobic bacterial counts (*P* < 0.001), with higher counts at Day 35 compared to Day 21 across all treatments (Fig. 1B). TB-300 treatment exhibited a significant increase in anaerobic bacterial counts from Day 21 to Day 35 (*P* = 0.046). There was no significant main effect of treatment (*P* = 0.353), indicating that average anaerobic counts did not differ among groups. Additionally, there was no significant interaction between age and treatment (*P* = 0.284), suggesting that the effect of treatment was consistent across both time points.

A significant interaction between age and treatment was observed for litter aerobic bacterial counts (*P* = 0.017) (Fig. 1C). At Day 21, there were no significant differences in aerobic counts among treatment groups (*P* > 0.05). However, by Day 35, birds in the SB-500 had significantly lower aerobic counts compared to the control (*P* = 0.023). Across all treatments, aerobic counts increased significantly from Day 21 to Day 35 (*P* < 0.0001), indicating that age had a strong effect within each treatment.

### Microbiological counts of cloacal and caecal content

**Fig. 2:**
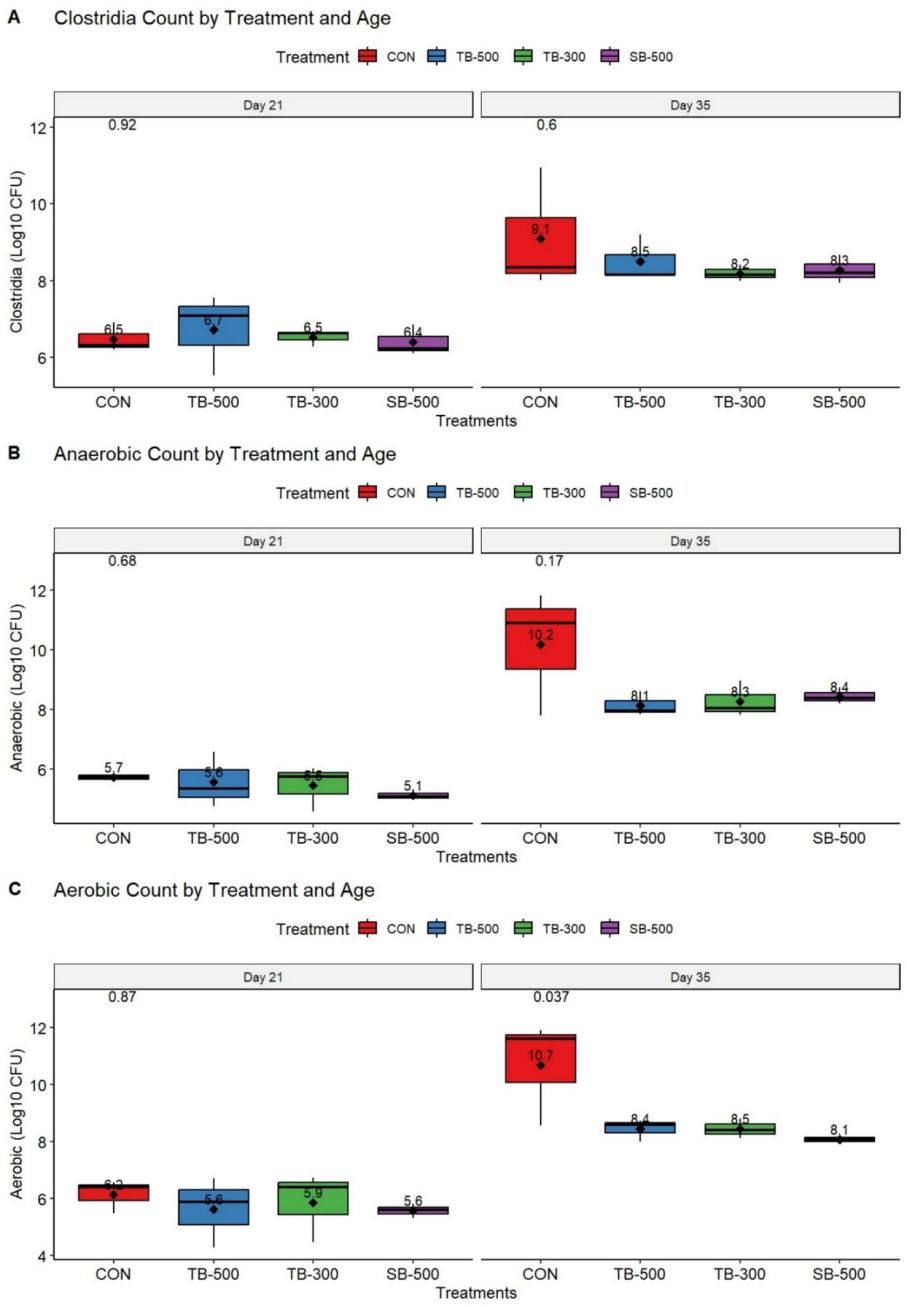
Impact of different dietary butyrate formulations on cloacal and cecal bacterial counts of broiler chickens at Days 21 and 35 of age. **CON**, Control basal diet; **TB-500**, basal diet + tributyrin 40%/copper/essential oils “Villa-Guard P™” (500g/ton feed); **TB-300**, basal diet + butyrins glycerol esters 60% “ProPhorce™ SR 130” (300g/ton feed); **SB-500**, basal diet + coated sodium butyrate 40% “Adimix® Precision” (500g/ton feed). Values of means were displayed above boxplots. Statistical significance was set at *P* ≤ 0.05. N = 6 birds per treatment.

Figure 2 presents the bacteriological load of cloacal swabs and caecal content on Days 21 and 35, respectively. Intestinal *Clostridia* counts were significantly affected by age (*P* < 0.001), with higher levels observed at Day 35 compared to Day 21 (Fig. 2A). However, there were no significant effects of treatment (*P* = 0.694) or age × treatment interaction (*P* = 0.688). Pairwise comparisons indicated a significant increase in counts from Day 21 to Day 35 in the control group (*P* = 0.011), but none of the treatments showed significant differences from control at either time point (*P* > 0.05).

Anaerobic bacterial counts were significantly affected by age (*P* < 0.001), with higher levels observed on Day 35 (Fig. 2B). No overall treatment effect (*P* = 0.116) or age × treatment interaction (*P* = 0.314) was found. However, post hoc comparisons revealed significant increases from Day 21 to Day 35 within all treatment groups, including TB-500 (*P* = 0.048), TB-300 (*P* = 0.025), and SB-500 (*P* = 0.007), as well as the control group (*P* < 0.001). These findings indicate a general age-related increase in anaerobic bacterial counts regardless of treatment.

Aerobic bacterial counts were significantly influenced by both age (*P* < 0.001) and treatment (*P* = 0.041), while the interaction between age and treatment was not significant (*P* = 0.236) (Fig. 2C). Post hoc pairwise comparisons indicated a marked increase in aerobic counts from Day 21 to Day 35 in the control group (*P* < 0.001), and a similar age-related increase was observed in TB-500 (*P* = 0.035). Day 35 aerobic counts were consistently higher in the control group than in most treatment groups at Day 21, suggesting a general age-related rise in bacterial load, modulated by treatment.

### Litter physical and chemical findings

**Fig. 3:**
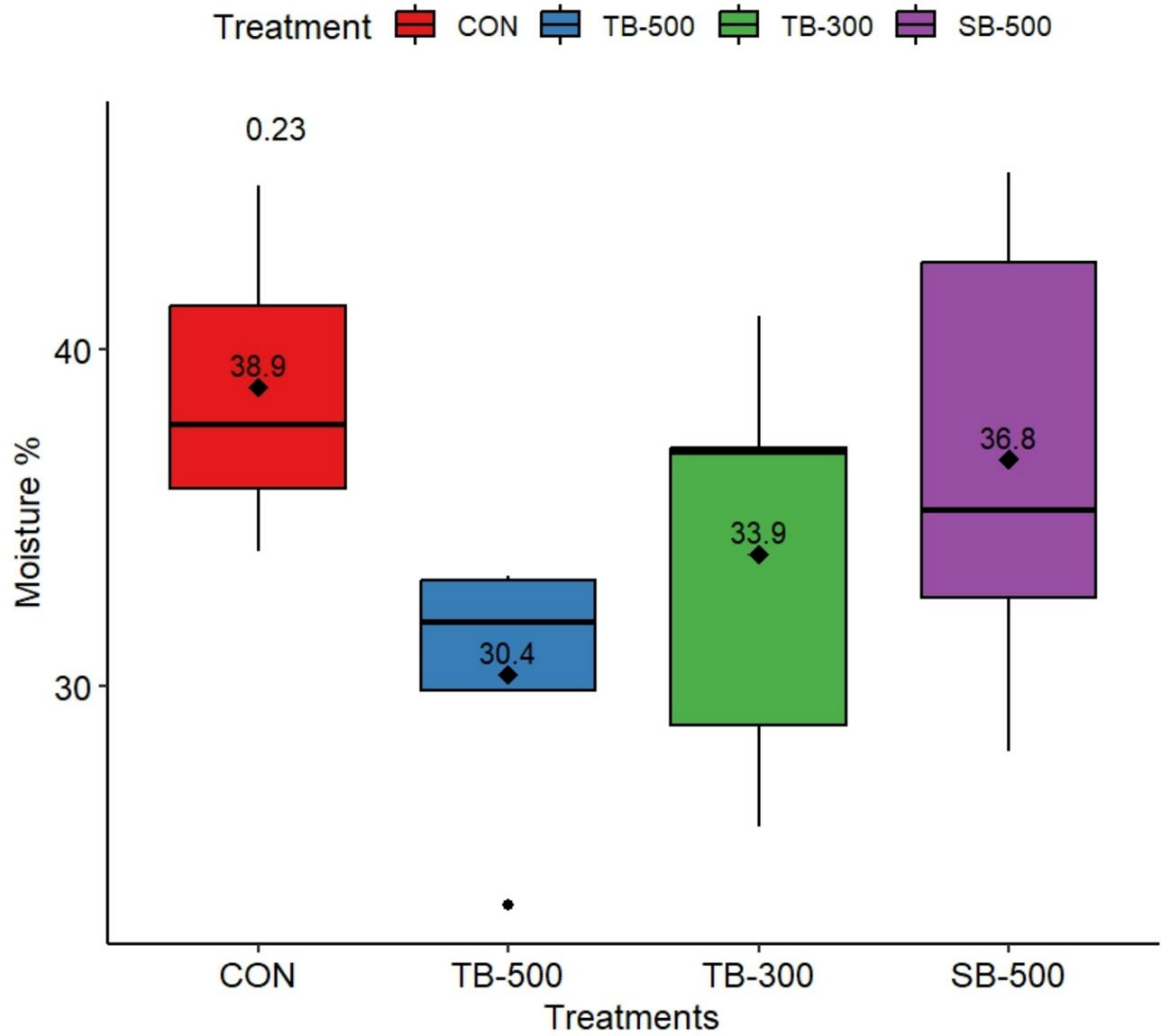
Impact of different dietary butyrate formulations on deep-litter moisture content (%) of broiler chickens at Day 35 of age. **CON**, Control basal diet; **TB-500**, basal diet + tributyrin 40%/copper/essential oils “Villa-Guard P™” (500g/ton feed); **TB-300**, basal diet + butyrins glycerol esters 60% “ProPhorce™ SR 130” (300g/ton feed); **SB-500**, basal diet + coated sodium butyrate 40% “Adimix® Precision” (500g/ton feed). Values of means were displayed inside boxplots. Statistical significance was set at *P* ≤ 0.05. N = 6 litter samples per treatment.

As shown in Fig. 3, the moisture content of deep litter on day 35 varied among the treatment groups. While the TB-500 group exhibited the lowest average moisture percentage, followed by TB-300 and SB-500, the differences were not statistically significant (*P* = 0.230). Regarding nitrogen content, all butyrate-supplemented groups (TB-500, TB-300, SB-500) showed notably higher total nitrogen levels in the litter (TB-500 = 2.21, TB-300 = 2.31, SB-500 = 2.23 g/kg) compared to the CON (1.70 g/kg).

### Histomorphometry of the small intestine

As detailed in Table 6, dietary supplementation significantly affected intestinal morphology on Day 35. In the duodenum, both TB-300 and SB-500 groups exhibited markedly enhanced villus length and crypt-to-villus (C/V) ratios, whereas the TB-500 group showed the deepest crypts compared to the CON group (*P* = 0.003). In the jejunum, villus length was significantly increased across all supplemented groups, with TB-500 showing the most pronounced improvement. Crypt depth was significantly greater in TB-500 and TB-300, while the SB-500 group had the highest C/V ratio (*P* = 0.001). In the ileum, the TB-500 group had the longest villi, and SB-500 achieved the highest C/V ratio. These enhancements suggest improved absorptive capacity and intestinal health due to butyrate supplementation.

**Table 6:** Impact of different dietary butyrate formulations on the histomorphometry of the small intestine of broiler chickens (mean µm ± SE) (Day 35).

Groups	Duodenum			Jejunum			Ileum		
	Villi length (µm)	Crypt depth (µm)	C/V^2^	Villi length (µm)	Crypt depth (µm)	C/V	Villi length (µm)	Crypt depth (µm)	C/V
CON	1559.80^b^	117.70^b^	13.49^ab^	1268.36^d^	107.57^b^	11.81^b^	932.16^ab^	112.48^a^	8.32^b^
TB-500	1622.73^b^	143.72^a^	11.46^b^	1637.45^a^	134.14^a^	12.18^b^	985.87^a^	106.91^a^	9.26^ab^
TB-300	1866.57^a^	121.69^b^	15.36^a^	1351.89^c^	124.95^a^	10.86^b^	947.38^ab^	106.49^a^	8.90^ab^
SB-500	1777.38^a^	113.07^b^	15.78^a^	1451.25^b^	104.82^b^	13.88^a^	885.98^b^	87.85^b^	10.14^a^
SEM^1^	31.32	3.57	0.48	32.56	3.34	0.32	13.44	2.56	0.22
*P*- value	** < 0.0001**	**0.003**	** < 0.001**	** < 0.0001**	** < 0.001**	**0.001**	**0.050**	** < 0.001**	**0.014**

Significant values are in bold.

^a,b,c,d^Mean showing variable letters within the same column denote statistical significance (Tukey’s test; *P* ≤ 0.05).

**CON**, Control basal diet; **TB-500**, basal diet + tributyrin 40%/copper/essential oils “Villa-Guard P™” (500g/ton feed); **TB-300**, basal diet + butyrins glycerol esters 60% “ProPhorce™ SR 130” (300g/ton feed); **SB-500**, basal diet + coated sodium butyrate 40% “Adimix® Precision” (500g/ton feed).

^1^SEM: Pooled standard error of the means.

^2^C/V: Crypt villus ratio.

N = Three intestinal sections × 5 birds per treatment.

### Blood biochemical indices

As shown in Table 7, dietary supplementation influenced several blood biochemical parameters. While albumin, triglycerides, creatinine, ALT, and amylase levels remained unaffected across groups, significant increases were observed in total protein, globulin, lipase, and protease levels in all supplemented groups compared to the CON. In contrast, total lipid, cholesterol, urea, and AST levels were significantly reduced, particularly in the TB-300 and SB-500 groups. These findings suggest improved metabolic and enzymatic profiles associated with the dietary treatments.

**Table 7:** Impact of dietary butyrate formulations on serum indices and serum activity of digestive enzymes of broiler chickens at Day 35.

Groups	Protein profile				Lipid profile			Liver functions		Kidney function		Serum activity of digestive enzymes		
	Total protein (g\dl)	Albumin (g\dl)	Globulin (g\dl)	A\G	Total lipids (g/dl)	Cholesterol (mg\dl)	TAG (mg\dl)	ALT (U\L)	AST (U\L)	Urea (mg/dl)	Creatinine (mg/dl)	Amylase (U/L)	Lipase (U/L)	Protease (U/L)
CON	5.08^b^	2.46	2.68^b^	0.93	197.40^a^	146.00^a^	58.00	31.80	86.25^a^	9.60^a^	0.30	117.40	168.00^b^	63.10^b^
TB-500	5.52^ab^	2.46	3.12^ab^	0.80	180.80^ab^	132.40^ab^	50.40	29.60	79.00^ab^	8.60^ab^	0.27	155.40	267.75^a^	86.04^a^
TB-300	6.00^a^	2.90	3.26^a^	0.89	165.80^b^	127.20^b^	44.60	24.00	79.00^ab^	6.50^b^	0.25	149.00	271.40^a^	89.52^a^
SB-500	6.06^a^	2.82	3.30^a^	0.86	164.60^b^	121.25^b^	43.00	24.60	69.40^b^	6.30^b^	0.27	143.25	274.25^a^	92.08^a^
SEM^1^	0.13	0.08	0.09	0.03	4.62	2.88	2.23	1.53	2.28	0.47	0.01	6.38	10.52	3.39
*P*- value	**0.015**	0.066	**0.047**	0.476	**0.024**	**0.006**	0.058	0.203	**0.027**	**0.018**	0.684	0.123	** < 0.0001**	**0.001**

Significant values are in bold.

^a,b^Mean showing variable letters within the same column denote statistical significance (Tukey’s test; *P* ≤ 0.05).

**CON**, Control basal diet; **TB-500**, basal diet + tributyrin 40%/copper/essential oils “Villa-Guard P™” (500g/ton feed); **TB-300**, basal diet + butyrins glycerol esters 60% “ProPhorce™ SR 130” (300g/ton feed); **SB-500**, basal diet + coated sodium butyrate 40% “Adimix® Precision” (500g/ton feed).

^1^SEM: Pooled standard error of the means.

A\G: Albumin\Globulin, TAG: Triacylglyceride, ALT: Alanine transaminase. AST: Aspartate transaminase.

N = 5 birds per treatment.

### Vaccinal antibody levels against NDV

The hemagglutination inhibition (HI) antibody titers against Newcastle disease virus showed average titers of 2.50 ± 0.67 log_2_ in CON, 4.00 ± 2.08 log_2_ in TB-500, 1.67 ± 1.20 log_2_ in TB-300, and 1.75 ± 0.63 log_2_ in SB-500. Although numerically higher titers were observed in TB-500 compared to CON and other treatment groups, the variability within treatments lacked statistical significance (*P* = 0.501).

### Expression of *mTOR*, *TLR4*, and *NBN* genes

**Fig. 4:**
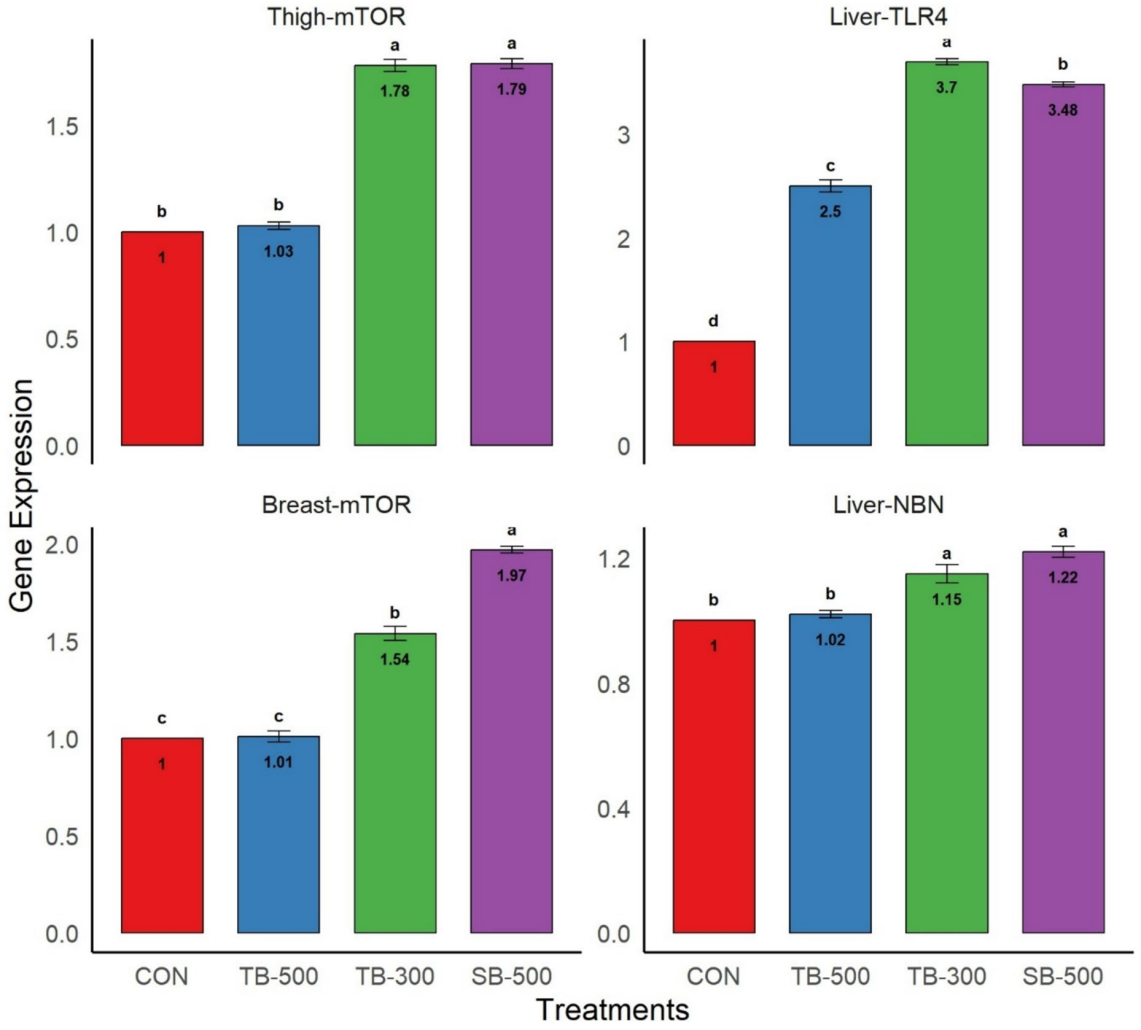
Impact of different dietary butyrate formulations on mRNA relative expression level for *mTOR* gene in breast and thigh muscles, as well as *TLR* and *NBN* genes in the liver of broiler chickens at Day 35 of age. **CON**, Control basal diet; **TB-500**, basal diet + tributyrin 40%/copper/essential oils “Villa-Guard P™” (500g/ton feed); **TB-300**, basal diet + butyrins glycerol esters 60% “ProPhorce™ SR 130” (300g/ton feed); **SB-500**, basal diet + coated sodium butyrate 40% “Adimix® Precision” (500g/ton feed). Data are represented as mean ± SE. Values of means were displayed inside bars. Groups having different letters are significantly different from each other at *P* ≤ 0.05. N = 5 birds per treatment.

The transcript levels of the *mTOR* gene in the breast and thigh muscles, as well as the liver *TLR4* and *NBN* genes of broiler chickens, are presented in Fig. 4. In the thigh muscle, *mTOR* expression was significantly upregulated in the TB-300 and SB-500 groups compared to both CON and TB-500 groups (*P* < 0.0001), while no significant difference was found between CON and TB-500 groups. Similarly, in the breast muscle, *mTOR* expression was highest in SB-500, followed by TB-300, both significantly higher than TB-500 and CON (*P* < 0.0001), which did not differ from each other. In the liver, *TLR4* gene expression was significantly upregulated in all butyrate-treated groups—TB-500, TB-300, and SB-500—compared to the control (*P* < 0.0001), with TB-300 showing the highest expression level. *NBN* gene expression in the liver was significantly elevated in TB-300 and SB-500 compared to both TB-500 and CON (*P* < 0.0001), while no difference was observed between TB-500 and CON.

## Discussion

Butyrate is a crucial short-chain fatty acid (SCFA) in the gastrointestinal tract. Among its derivatives, sodium butyrate and tributyrin, each offering distinct but beneficial effects on broiler growth and health^44^. The present study demonstrated that dietary supplementation with butyrate derivatives, particularly butyrate glycerol esters (TB-300) and coated sodium butyrate (SB-500), significantly enhanced broiler growth performance. Coated sodium butyrate 40% at 500 g/ton increased body weight during the fourth week. However, on Day 35, coated sodium butyrate (SB-500) exhibited a significantly higher FCR compared to glycerol esters of butyric acid supplements (TB-300 and TB-500), indicating less efficient feed conversion despite improved BW. These finding aligns with^44^, who reported that 0.6 g/kg of sodium butyrate enhanced average daily weight gain during the first 21 days of broiler growth. Similarly, another study showed that sodium butyrate increased pancreatic size and enzyme activity, thereby improving digestion and nutrient absorption, which contributed to enhanced growth performance^45^. Consistent with this, Hassan et al.^45^ observed a dose-dependent increase in body weight gain with 500–2000 mg/kg sodium butyrate supplementation in broilers. Hu and Guo^46^ noted that uncoated sodium butyrate is quickly absorbed in the upper gut, limiting its efficacy, which led to the development of coated forms for sustained intestinal release. They further reported that 800 mg/kg of coated sodium butyrate improved FCR in layers by enhancing gut morphology and nutrient utilization. However, some studies have shown no significant effects on growth or FCR^47,48^, likely due to differences in bird age, breed, health status, dosage, or hygiene conditions.

Notably, in the current study, the TB-300 group exhibited consistent improvements across performance indicators—higher BW, lower FCR, and superior efficiency—supporting the potential of tributyrin glycerol esters as an effective additive for broiler production, especially in the later growth phases. Di- and tri-butyrin 60% (TB-300) supplementation at 300 g/ton led to a significant increase in body weight in the fifth week, and resulted in the lowest mortality rate, as well as the highest European Production Efficiency Factor (EPEF). The tributyrin/copper/essential oils mixture 40% (TB-500) at 500 g/ton showed marginal improvements in growth performance parameters compared to the control. On Day 35, both TB-300 and TB-500 showed significantly lower FCR values than the control group, suggesting better nutrient utilization. Importantly, feed intake remained largely unaffected across all butyrate-supplemented groups, indicating that the improvements in growth metrics were due to enhanced feed efficiency rather than increased consumption. Pires et al.^48^ reported that tributyrin supplementation increased final body weight in broilers by enhancing the abundance of SCFA-producing bacteria and stimulating butyrate production in the intestinal tract. The present results also support findings by^48^ and^74^  , who observed that tributyrin supplementation lowered broiler FCR. Furthermore, another study demonstrated that dietary inclusion of butyrate glycerides at levels ranging from 2000 to 10,000 mg/kg improved final body weight and FCR in broilers^49^.

The inclusion of butyrate derivatives in broiler diets did not significantly influence carcass yield or the relative weights of carcass parts. While some studies have reported improved carcass yield with butyrate salts, these effects were observed when combined with antioxidants or synbiotics^50,51^. In contrast, the current findings are consistent with reports indicating no significant impact from 1 g/kg of protected sodium butyrate on carcass traits^47^. However, other research has shown enhanced carcass yield in broilers supplemented with tributyrin^11^.

Supplementing broiler diets with butyrate derivatives (TB-500, TB-300, SB-500) effectively reduced microbial counts in the large intestine and deep litter. Notably, birds receiving coated sodium butyrate (SB-500) exhibited significantly lower aerobic and clostridial bacterial loads in deep litter by Day 35. These findings align with^53^  , who reported that protected sodium butyrate has superior antibacterial effects due to its sustained intestinal release. Supporting this, Hassan et al., ^45^ found that SB lowered intestinal pH, thereby reducing pathogen colonization, and ^75^ demonstrated its effectiveness in reducing caecal Enterobacteriaceae counts. The antimicrobial action of sodium butyrate is attributed to its conversion to butyric acid, which disrupts bacterial cell pH and enzyme function, leading to cell death^54^. Additionally, Bedford and Gong^1^ reported that butyrate glycerides shift caecal microbiota toward beneficial, butyrate-producing bacteria that enhance host metabolism. Tributyrin, as noted by ^74^ and ^67^, also reduced coliform counts and modulated the microbial community, further supporting its role as an effective antibiotic alternative.

Dietary supplementation with butyrate derivatives influenced litter moisture quality, particularly in broilers receiving glycerol esters of butyric acid (TB-500 and TB-300), followed by coated sodium butyrate (SB-500). This improvement may be linked to sodium butyrate’s ability to enhance intestinal barrier function^55^ by reducing permeability through upregulation of epithelial tight junction proteins like occludin^56^. Nitrogen levels in deep litter were slightly higher in all butyrate-supplemented groups compared to the control, though differences were minimal. This aligns with previous findings by^3^, who reported similar or slightly elevated litter nitrogen levels with buffered sodium butyrate supplementation. In contrast, another study on ducks showed that 700 mg/kg of sodium butyrate reduced fecal nitrogen, phosphorus, and ammonia emissions, highlighting variability in environmental outcomes depending on species and conditions^57^.

Small intestinal histomorphometry was significantly influenced by dietary butyrate additives. The TB-500 group (tributyrin/copper/essential oils mixture) showed the deepest duodenal and jejunal crypts and the longest villi in the jejunum and ileum. TB-300 (di- and tri-butyrin) yielded the longest duodenal villi and deepest jejunal crypts, with a duodenal crypt-to-villus ratio and jejunal villus length higher than the control but lower than the SB-500 group. These results align with previous studies showing enhanced crypt depth and epithelial turnover in broilers supplemented with 2000 mg/kg butyrate glycerides. Hu et al.^67^ also reported that tributyrin has a longer half-life and greater systemic absorption, contributing to superior intestinal development and heavier jejune compared to sodium butyrate.

Coated sodium butyrate (SB-500) significantly enhanced duodenal and jejunal villi lengths and crypt-to-villus (C/V) ratios. These findings align with Hu & Guo^46^, who observed increased DNA, RNA, and protein content in the duodenal mucosa with rising sodium butyrate doses (500–2000 mg/kg), suggesting enhanced mucosal development. Similarly, Zhang et al.^58^ reported that protected sodium butyrate led to reduced jejunal crypt depth and increased C/V ratio. Additionally, the results are consistent with the findings of^46^ , who reported that increasing the inclusion rate of sodium butyrate in the poultry diet expanded the jejunal C/V ratio accordingly. The improved intestinal architecture may result from butyrate’s stimulation of blood circulation and peptide secretion, promoting enterocyte proliferation, mucosal repair, and villus elongation^59^. Supporting this, prior studies noted that sodium butyrate supplementation upregulates gene expression, boosts protein synthesis, increases intestinal segment size and weight, enhances epithelial cell proliferation, and reduces enterocyte apoptosis^45,60^.

Supplementation with glycerol esters of butyric acid (TB-300) and coated sodium butyrate (SB-500) significantly improved blood biochemical parameters in broilers, notably increasing serum total proteins, albumins, and globulins. Elevated serum albumin, a marker of nutritional status^61^, has been positively associated with higher body weights^62^. These findings align with previous studies^11,59^, which linked glyceryl butyrate or sodium butyrate supplementation to enhanced protein levels due to improved enzyme activity, pancreatic secretion, nutrient digestibility, and absorption. However, contrasting results were noted by a study reporting reduced protein levels with 1 g/kg of 40% sodium butyrate in feed^51^.

Di- and tri-butyrin (TB-300) and coated sodium butyrate (SB-500) significantly reduced serum lipid parameters—including total lipids, cholesterol, and triglycerides—while tributyrin/copper/essential oil mixture (TB-500) showed a numerical reduction. These findings align with earlier studies reporting that butyrate glycerides lower serum lipid levels by inhibiting intestinal lipid synthesis and promoting hepatic lipid oxidation^11,63^. Tributyrin has also been linked to reduced fat deposition in broiler tissues^44^. Furthermore, Bedford and Gong^1^ stated that butyrate glycerides have been shown to modulate cecal microbiota, enhancing lipid metabolism through microbial-derived metabolites such as choline and lactate. Also, sodium butyrate was previously proven to reduce serum cholesterol^59^. The hypolipidemic effect of SCFAs was attributed to their prolonged suppressive effect on the hepatic 3-hydroxy-3-methylglutaryl-CoA reductase and synthase^64^.

In this study, dietary supplementation with butyrate formulations (TB-500, TB-300, SB-500) improved liver and kidney functions in broilers, as evidenced by reduced serum ALT, AST, and urea levels. Coated sodium butyrate significantly lowered AST, consistent with ^59^, while tributyrin (TB-300) and SB-500 also reduced ALT, supporting the hepatoprotective role noted by^74^ . Additionally, TB-300 and SB-500 improved renal function by decreasing blood urea, aligning with findings that tributyrin reduces serum creatinine and uric acid, indicating reduced oxidative stress^74^ . However, some studies have reported no significant effects of butyrate derivatives on these biochemical markers^65^. The three dietary butyrate supplements (TB-500, TB-300, SB-500) significantly enhanced serum digestive enzyme activity (lipase and protease), supporting improved digestive function. These findings align with^74^ , who reported that butyrate and its derivatives stimulate pancreatic activity and elevate the secretion of digestive enzymes like amylase and lipase.

Dietary supplementation with the tributyrin/copper/essential oils mixture (TB-500) slightly increased antibody titers against NDV, although no significant association was found between butyrate supplementation and vaccinal antibody levels. This aligns with previous studies reporting inconsistent effects of butyrate on humoral immunity, some showing no significant impact^3,66^, while others demonstrated improvements^67^.

Dietary supplementation with di- and tri-butyrin (TB-300) and coated sodium butyrate (SB-500) significantly upregulated *mTOR* gene expression in broiler muscle tissue, with SB-500 producing the highest transcript levels in breast muscle. The *mTOR* gene encodes a kinase involved in regulating muscle growth, protein synthesis, and cellular metabolism. Its upregulation supports skeletal muscle development and enhances nutrient utilization and regeneration of intestinal epithelial cells^68–71^. These findings align with previous studies reporting the stimulatory effect of sodium butyrate on growth-related gene expression in poultry^3^. All three dietary butyrate supplements (TB-500, TB-300, SB-500) also significantly upregulated *TLR4* gene expression in the broilers’ liver, with the highest expression observed in TB-300. *TLR4*, a key innate immunity gene, plays a central role in pathogen recognition, immune signalling, and linking innate and adaptive immune responses^71^. These results align with prior studies reporting sodium butyrate’s ability to enhance *TLR4* expression in poultry liver^3^ and with findings by^11^, who noted increased lymphoid organ weights with tributyrin supplementation. Furthermore, dietary supplementation with glycerol esters of butyric acid (TB-300) and coated sodium butyrate (SB-500) significantly upregulated *NBN* gene expression in the broilers’ liver. The *NBN* gene, associated with DNA repair and genomic stability, encodes a protein that facilitates detection and repair of DNA double-strand breaks by coordinating the MRN complex (MRE11A, RAD50). This upregulation suggests enhanced cellular stress response and maintenance of chromosomal integrity in butyrate-supplemented birds^71^.

## Conclusions

Dietary supplementation with butyrate derivatives improved broiler performance, intestinal morphology, microbial balance, and gene expression profiles. Among the tested formulations, glycerol esters of butyric acid (TB-300) consistently showed the most favorable effects, including enhanced body weight, feed efficiency, intestinal structure, and upregulation of growth- (*mTOR*), immune- (*TLR4*), and DNA repair-related (*NBN*) genes. Coated sodium butyrate (SB-500) also demonstrated notable benefits, particularly in reducing litter bacterial load and enhancing *mTOR* and *NBN* expression. These findings support the use of specific butyrate derivatives as functional feed additives to optimize poultry growth and health.

## Data Availability

The data supporting this study’s findings are available from the corresponding author upon reasonable request.

## Abbreviations

ALT: Alanine aminotransferase
ANOVA: Analysis of variance
AST: Aspartate aminotransferase
BW: Body weight
CFU: Colony-forming units
EPEF: European production efficiency factor
FCR: Feed conversion ratio
FI: Feed intake
HI: Haemagglutination inhibition
*mTOR*: Mechanistic target of rapamycin
*NBN*: Nibrin
NDV: Newcastle disease virus
SB: Sodium butyrate
SBM: Soybean meal
SCFAs: Short-chain fatty acids
TAG: Triglycerides
TB: Tributyrin
*TLR*: Toll-like receptors
